# Efficacy of the posterior nasal nerve resection combined with hormone transnasal nebulization on difficult-to-treat rhinosinusitis: a retrospective analysis

**DOI:** 10.1016/j.bjorl.2024.101413

**Published:** 2024-03-01

**Authors:** Xin-ke Wang, Qi-ling Zheng, Jia-ning Sun

**Affiliations:** a The Third Hospital of Ninghai County, Department of Otorhinolaryngology, Ningbo, China; b Yuyao People's Hospital of Zhejiang Province, Department of Otolaryngology Head and Neck Surgery, Yuyao, China

**Keywords:** Posterior nasal nerve (PNN) resection, Budesonide suspension, Transnasal nebulization, Difficult-to-treat rhinosinusitis (DTRS)

## Abstract

**Objective:**

A retrospective analysis was performed to explore the clinical effect of the Posterior Nasal Nerve (PNN) resection combined with hormone transnasal nebulization on Difficult-to-Treat Rhinosinusitis (DTRS).

**Methods:**

A total of 120 DTRS patients were selected and divided into a control group (*n* = 60) and a study group (*n* = 60) according to different treatments. The control group patients were treated via PNN resection, followed by normal saline transnasal nebulization; the study group patients were given PNN resection and then treated with budesonide suspension transnasal nebulization. Subsequently, the comparison was performed between the two groups in terms of (1) Clinical baseline characteristics; (2) Sino-nasal Outcome Test (SNOT)-22 scores before treatment and after 3-months, 6-months and 12-months of treatment; (3) Lund-MacKay scores before treatment and after 10, 30, 90, and 180 days of treatment; (4) Incidence of adverse reactions during treatment.

**Results:**

There was no significant difference in SNOT-22 or Lund-Kennedy scores between the two groups before treatment (*p* > 0.05). After treatment, the SNOT-22 and Lund-Kennedy scores of the control and the study groups were decreased, and compared with the control group, the SNOT-22 and Lund-Kennedy scores in the study group improved more significantly (*p* < 0.05). In addition, the study group and the control group presented with 1 and 4 cases of nasal adhesion, 2 and 3 cases of epistaxis, 1 and 4 cases of sinus orifice obstruction, 1 and 3 cases of lacrimal duct injuries, respectively. The incidence of adverse reactions in the study group was significantly lower than that in the control group (8.3% vs. 23.3%) (*p* < 0.05).

**Conclusion:**

PNN resection combined with hormone transnasal nebulization treatment can improve the symptoms and quality of life of DTRS patients, with good clinical efficacy but few adverse reactions. Therefore, such combination treatment deserves a promotion and application clinically.

**Level of Evidence:**

Level 3.

## Highlights

• PNN resection & hormone transnasal nebulization shows good efficacy on DTRS.
• Combination can relieve the symptoms of DTRS patients, reduce adverse reactions.
• The combination deserves a promotion in clinical practice.

## Introduction

Chronic Rhinosinusitis (CRS) is a group of chronic inflammatory diseases that frequently occur in the nasal cavity and sinus mucosa.^1^ As one of the most common conditions in otorhinolaryngology, CRS is characterized by multiple occurrences and recurrence. In addition, the morbidity of CRS is rising year by year, and about 8% of incidence of CRS has been reported in China.^2^, ^3^ Difficult-to-Treat Rhinosinusitis (DTRS) is a subtype of CRS. As described by the European Position Paper on Rhinosinusitis and Nasal Polyps 2012, DTRS can be diagnosed if a CRS patient's symptoms are not still controlled after surgery, 1-year of nasal glucocorticoid treatment and 2 courses of systemic glucocorticoid or antibiotic treatment.^4^ Despite treatment by drugs and surgery, DTRS patients are still affected by clinical symptoms such as purulent nasal discharge, nasal obstruction, olfactory dysfunction or facial pain that occur repeatedly, seriously affecting their physical and mental health and quality of life. Moreover, long-term treatment also brings a huge financial burden to patients.^5^ Therefore, there is an urgent need to find a more efficient and feasible treatment for improving the outcomes of DTRS patients.

Endoscopic Posterior Nasal Nerve (PNN) resection, a highly selective vidian neurectomy, can inhibit secretion-promoting movement and suppress neurogenic inflammation induced by parasympathetic and sensory nerves. In recent years, endoscopic PNN has been commonly used to reduce the sensitivity of nasal mucosa in patients with allergic rhinitis and improve their clinical symptoms.^6^, ^7^ Endoscopic surgery has the advantages of providing a clear field of view and easy removal of lesions and has been highly accepted by most patients and their families in clinical practice.^8^ However, the efficacy of surgical treatment is expected to be reserved for cases where anatomical/functional causes are identified that can be addressed by it, but in no way will it interrupt the course of the disease or its propensity to develop comorbidities. Drug treatment can relieve tissue edema and inflammation in patients, reduce the difficulty of endoscopic surgery, depress the recurrence of postoperative symptoms, and provide great help to control the patient's condition.^9^, ^10^ It is reported that surgery combined with drugs can more effectively improve clinical treatment outcomes.^11^

The important pathological basis of DTRS is mainly inflammation, as increased levels of pro-inflammatory factors accelerate the progression of the disease and further aggravate the damage to the normal tissues of the nasal mucosa. Hence, anti-inflammatory therapy could be the key treatment direction of DTRS.^10^, ^11^ In addition, nasal glucocorticoids can effectively alleviate and inhibit inflammatory response and reduce postoperative adverse reactions, thereby improving clinical efficacy. Nevertheless, the curative effect of glucocorticoids may be affected if they cannot completely reach and cover the surgical wound due to improper use by postoperative patients.^12^ Budesonide suspension, a respiratory tract nebulized inhaled steroid hormone, belongs to strong anti-inflammatory and anti-allergic drugs. Generally, budesonide suspension can inhibit the activation of various inflammatory cells in the nasal cavity, alleviate the inflammation damage of lesion sites and promote the absorption of mucosal edema, thereby accelerating the recovery of nasal function and relieving the symptoms such as nasal congestion and purulent nasal discharge.^13^ Currently, there are few studies on the comprehensive efficacy of PNN resection combined with budesonide suspension in treating DTRS. Therefore, a retrospective analysis was performed to explore the clinical effect of PNN resection combined with budesonide suspension on DTRS patients in this study.

## Methods

### Study subjects

We conducted a study involving 120 DTRS patients who received treatment at the Department of Otorhinolaryngology-Head and Neck Surgery in our hospital from July 2017 to October 2021. All participants in this study provided informed consent, and the study received approval from our hospital's ethics committee. The patients were then divided into two groups: a control group (*n* = 60), which underwent PNN resection, and a study group (*n* = 60), where patients received a combination of PNN resection and budesonide suspension transnasal nebulization. The detailed clinical data for all subjects were collected.

Inclusion and exclusion criteria were shown as follows. Patients were included in this study if they (1) Satisfied the diagnosis standard of DTRS^4^; (2) Were aged 18-years or older; (3) Underwent nasal endoscopy and three-dimensional CT examinations of the paranasal sinuses; and (4) Had not received antihistamines, glucocorticoids, or immunosuppressive agents within one month before the surgery.^14^ Exclusion criteria encompassed patients who (1) Had serious internal or surgical conditions, such as tuberculosis, hepatitis, hypertension, diabetes, or malignant tumors; (2) Had systemic inflammatory diseases, ciliary motility disorders, or cystic fibrosis; (3) Were pregnant or lactating; or (4) Were unable to complete questionnaires or clinical assessments due to psychiatric disorders.

### Therapeutic methods

Both groups underwent PNN resection as part of their treatment protocol. After the surgical procedure, the control group received transnasal nebulization with normal saline, while the study group received budesonide suspension transnasal nebulization.

PNN resection: The patient was positioned supine, followed by routine intubation under general anesthesia. Epinephrine saline, with a ratio of 1:1000, was applied to contract the nasal mucosa. Subsequently, a longitudinal incision, approximately 0.5 cm in front of the tail of the middle turbinate, was made under nasal endoscopy to expose the ethmoidal crest of the palatine bone. After removing the ethmoidal crest using bone-biting forceps, the neurovascular bundle, which includes the PNN and the sphenopalatine artery along with their branch vessels, was carefully separated from the sphenopalatine foramen. Particular attention was given to avoiding damage to blood vessels and preventing bleeding during the procedure. Following this, the PNN was excised, and medical cotton balls were used to achieve full hemostasis within the surgical cavity. The mucosal valve of the middle nasal meatus was then reset. The middle nasal meatus was filled with an expanding sponge, and intravenous antibiotics were administered for 1–2 days post-surgery. Three days later, the sponge was removed.

Normal saline transnasal nebulization: Patients in the control group received daily transnasal nebulization with 5 mL of normal saline, with each session lasting 5 min. This nebulization therapy was continued for a duration of 14 days.

Hormone transnasal nebulization: Patients in the study group underwent bilateral nasal nebulization inhalation of budesonide suspension (0.5 mg/mL per dose, AstraZeneca Pty Ltd. Australia, H20140475) once a day, with each session lasting 5 min. This treatment was also administered for a total of 14 days.

### Outcome measures

(1) Sino-nasal Outcome Test (SNOT-22) scoring: The SNOT-22 questionnaire was used to assess symptom improvement in both groups before treatment and at 3-months, 6-months, and 12-months after treatment. This evaluation encompassed five categories: rhinology, extranasal symptoms, ear/face symptoms, psychological dysfunction, and sleep dysfunction, with each category containing multiple items. Based on individual patient circumstances, these items were categorized as none, occasional, rare, usual, many, or frequent, with corresponding scores of 0–5 assigned, respectively.^15^ Higher scores indicated more severe symptoms. The changes in SNOT-22 scores before and after treatment were compared between the two groups.
(2) Lund-Kennedy scoring: Patients underwent nasal endoscopy, and their conditions were assessed according to the Lund-Kennedy scoring criteria. This assessment included five dimensions: nasal polyps, mucosal edema, presence or absence of secretions, scars, and scabbing, both before treatment and at 10-days, 30-days, 90-days, and 180-days after treatment.^16^ The scoring criteria were as follows: a) Nasal polyps: 0 = no polyps, 1 = polyps only present in the middle nasal tract, 2 = polyps extending beyond the middle nasal tract. b) Edema: 0 = none, 1 = mild, 2 = severe. c) Secretions: 0 = no secretions, 1 = clear secretions and thin rhinorrhea, 2 = viscous secretions and purulent rhinorrhea. d) Scars: 0 = none, 1 = mild, 2 = severe. e) Scabbing: 0 = none, 1 = mild, 2 = severe. The changes in Lund-Kennedy scores before and after treatment were compared between the two groups.
(3) Adverse Reactions: Adverse reactions occurring during the treatment period, including nasal adhesion, epistaxis, sinus orifice obstruction, and lacrimal duct injuries, were compared between the two groups.

### Statistical analysis

Sample size calculation was conducted using GPower software, with the primary outcome being the Lund-Kennedy scoring. The effect size of budesonide suspension transnasal nebulization treatment on Lund-Kennedy scoring was determined based on previous case data. To achieve a statistical power of 80% and a two-tailed alpha error of 0.05, a total sample size of 120 patients, with 60 patients in each group, was estimated to be required.

Data analysis was performed using SPSS 19.0 statistical software. Descriptive statistics for measurement data were presented as mean ± standard deviation, and group comparisons were conducted using the *t*-test. Enumeration data were presented as n (%) and compared among groups using the Chi-Squared (χ²) test. A significance level of *p* < 0.05 was considered to indicate a statistically significant difference.

## Results

### Clinical characteristics of the two group patients

As shown in Table 1, a total of 120 patients were enrolled and divided into a control group (*n* = 60) and a study group (*n* = 60). The control group consisted of 30 males and 30 females; the age was 36–76 years old, and the mean age was 57.60 ± 10.4 years old; the course of the disease was 1–7 years, and the mean course of the disease was 4.05 ± 1.31 years. In the study group, there were 35 cases of males and 25 cases of females, with an age distribution of 36–78 years old and mean age of 56.82 ± 10.83 years old; besides, the course of disease was 1–7 years, and the mean course of disease was 4.22 ± 1.31 years. There were no significant differences between the two groups of patients in terms of the clinical data (age, gender, history of smoking, course of disease, complications, and Lund-Mackay scores) (*p* > 0.05). Therefore, the two group patients were comparable.

**Table 1:** Clinical characteristics of the patients in both groups.

	Study group (*n* = 60)	Control group (*n* = 60)	χ²/t	*p*
Age (year)	56.82 ± 10.83	57.60 ± 10.4	−0.404	0.687
Gender (%)			0.839	0.360
Male	35 (58.3)	30 (50.0)		
Female	25 (41.7)	30 (50.0)		
History of smoking (%)			0.853	0.356
No	28 (46.7)	23 (38.3)		
Yes	32 (53.3)	37 (61.7)		
Course of disease (year)	4.22 ± 1.35	4.05 ± 1.31	0.686	0.494
Complication (%)				
Asthma (%)	8 (13.3)	6 (10.0)	0.323	0.570
Obstructive sleep apnea (%)	1 (1.7)	0 (0)	–	1.000
History of depression (%)	12 (20.0)	8(13.3)	0.96	0.327
Nasal polyp (%)	2 (3.3)	4 (6.7)	0.175	0.675
Otitis media (%)	25 (41.7)	31 (51.7)	1.205	0.272
Lund-Mackay score	8.72 ± 1.46	8.78 ± 1.49	−0.248	0.805

Note: “-” indicated no exact value; the measurement data were expressed by ($\bar{\text{X}}\pm \text{S}$); the enumeration data were expressed as *n* (%).

### Comparison of Sino-nasal Outcome Test (SNOT-22) scores between the two groups before and after treatment

After comparison, the five SNOT-22 scoring items of the two groups, including nasal symptoms, extranasal symptoms, ear/face symptoms, sleep dysfunction and psychological dysfunction, were not significantly different before treatment (*p* > 0.05) (Table 2). Five-item scores decreased in both groups after 3-, 6-, and 12-months of treatment. Upon 3-months of treatment, compared with the control group, the study group exhibited lower scores of nasal symptoms, ear/face symptoms and sleep dysfunction, and the difference was statistically significant (*p* < 0.05); however, the differences in scores of extranasal symptoms and psychological dysfunction between the two groups were not statistically significant (*p* < 0.05). Additionally, 6- and 12-months of treatment later, the patients in the study group showed lower scores of nasal symptoms, extranasal symptoms, ear/facial symptoms, sleep dysfunction and psychological dysfunction compared with those in the control group, and the differences were statistically significant (*p* < 0.05).

**Table 2:** Comparison of Sino-nasal Outcome Test (SNOT-22) scores between the two groups before and after treatment.

	Grouping	*n*	T0	T1	T2	T3
Nasal symptoms	Study group	60	17.48 ± 2.00	14.40 ± 1.82	10.38 ± 1.76	6.30 ± 1.59
	Control group	60	17.98 ± 2.00	15.30 ± 1.92	12.08 ± 1.91	8.90 ± 1.45
	*t*		−1.366	−2.635	−5.078	−9.380
	*p*		0.174	0.010	<0.001	<0.001
Extranasal symptoms	Study group	60	7.78 ± 1.88	6.38 ± 1.44	4.78 ± 1.04	3.00 ± 0.96
	Control group	60	7.73 ± 1.65	6.90 ± 1.47	6.05 ± 1.03	4.63 ± 1.02
	*t*		0.155	−1.946	−6.687	−9.026
	*p*		0.877	0.054	<0.001	<0.001
Ear/facial symptoms	Study group	60	9.93 ± 1.83	8.28 ± 1.70	6.33 ± 1.47	5.30 ± 1.14
	Control group	60	9.78 ± 1.98	9.03 ± 1.50	8.02 ± 1.60	7.18 ± 1.35
	*t*		0.431	−2.567	−6.003	−8.27
	*p*		0.667	0.011	<0.001	<0.001
Sleep dysfunction	Study group	60	10.87 ± 2.19	8.73 ± 1.51	6.47 ± 1.68	4.05 ± 1.20
	Control group	60	10.60 ± 2.06	9.42 ± 1.94	7.58 ± 1.48	5.85 ± 1.18
	*t*		0.687	−2.154	−3.865	−8.300
	*p*		0.493	0.033	<0.001	<0.001
Psychological dysfunction	Study group	60	12.75 ± 2.18	9.48 ± 1.65	6.20 ± 1.35	3.08 ± 1.00
	Control group	60	12.42 ± 1.83	10.07 ± 1.61	8.45 ± 1.06	6.07 ± 1.01
	*t*		0.907	−1.956	−10.136	−16.319
	*p*		0.366	0.053	<0.001	<0.001

Note: T0, before treatment; T1, after 1-months of treatment; T2, after 6-months of treatment; T3, after 12-months of treatment; the measurement data were expressed by ($\bar{\text{X}}\pm \text{S}$).

### Comparison of Lund-Kennedy scores between the two groups before and after treatment

Before treatment, the Lund-Kennedy scores of nasal polyps, mucosal edema, secretion, scar, and scabbing between the two groups were not statistically significant (*p* > 0.05) (Table 3). At 10-, 30-, 90- and 180-days after treatment, the five-dimensional scores of patients in both groups decreased. At 10 days after treatment, patients in the study group had lower scores of scabbing compared with the control group, with statistically significant differences (*p* < 0.05); while there were no significant differences between the two groups in scores of nasal polyps (*p* > 0.05), mucosal edema (*p* > 0.05), secretions (*p* > 0.05), and scars (*p* = 0.05). At 30-days after treatment, the study group patients presented lower scores of nasal polyps, secretions, scars, and scabbing than the control group patients, with significant differences (*p* < 0.05); while in comparison of mucosal edema scores, the differences between the two groups were not statistically significant (*p* > 0.05). At 90- and 180-days after treatment, the study group patients exhibited lower scores of nasal polyps, mucosal edema, secretions, scars, and scabbing compared with the control group patients, all with significant differences (*p* < 0.05).

**Table 3:** Comparison of Lund-Kennedy scores before and after treatment between the two groups.

	Grouping	*n*	Before treatment	After treatment			
				10-days	30-days	90-days	180-days
Nasal polyps	Study group	60	1.78 ± 0.61	1.42 ± 0.56	1.08 ± 0.56	0.87 ± 0.50	0.70 ± 0.56
	Control group	60	1.70 ± 0.53	1.50 ± 0.50	1.33 ± 0.48	1.08 ± 0.42	0.98 ± 0.47
	*t*		0.796	−0.856	−2.633	−2.552	−3.000
	*p*		0.428	0.394	0.010	0.012	0.003
Mucosal edema	Study group	60	1.45 ± 0.50	1.20 ± 0.48	0.93 ± 0.52	0.63 ± 0.49	0.48 ± 0.54
	Control group	60	1.48 ± 0.50	1.33 ± 0.48	1.10 ± 0.48	0.92 ± 0.42	0.82 ± 0.50
	*t*		−0.363	−1.529	−1.837	−3.405	−3.508
	*p*		0.717	0.129	0.069	0.001	0.001
Secretions	Study group	60	1.15 ± 0.44	0.92 ± 0.28	0.68 ± 0.50	0.55 ± 0.50	0.33 ± 0.48
	Control group	60	1.13 ± 0.34	1.02 ± 0.39	0.88 ± 0.42	0.78 ± 0.42	0.63 ± 0.49
	*t*		0.23	−1.615	−2.372	−2.775	−3.418
	*p*		0.818	0.109	0.019	0.006	0.001
Scars	Study group	60	1.42 ± 0.50	1.15 ± 0.36	0.78 ± 0.42	0.63 ± 0.49	0.40 ± 0.49
	Control group	60	1.45 ± 0.50	1.30 ± 0.46	1.10 ± 0.35	0.93 ± 0.36	0.87 ± 0.39
	*t*		−0.366	−1.983	−4.493	−3.835	−5.748
	*p*		0.715	0.05	<0.001	<0.001	<0.001
Scabbing	Study group	60	1.38 ± 0.49	1.15 ± 0.36	0.98 ± 0.22	0.73 ± 0.45	0.62 ± 0.49
	Control group	60	1.37 ± 0.49	1.35 ± 0.48	1.20 ± 0.44	1.17 ± 0.42	1.08 ± 0.33
	*t*		0.187	−2.578	−3.376	−5.489	−6.093
	*p*		0.852	0.011	0.001	<0.001	<0.001

Note: The measurement data were expressed by ($\bar{\text{X}}\pm \text{S}$).

### Comparison of adverse reactions between the two groups during treatment

As presented in Table 4, 1 and 4 cases of nasal adhesion, 2 and 3 cases of epistaxis, 1 and 4 cases of sinus orifice obstruction, 1 and 3 cases lacrimal duct injuries could be observed in the study group and the control group, respectively. In comparison with the control group (23.3%), the incidence of adverse reactions in the study group (8.3%) was significantly lower (*p* < 0.05).

**Table 4:** Comparison of adverse reactions between the two group patients.

	Study group	Control group	χ²	*p*
Nasal adhesion (%)	1 (1.7)	4 (6.7)		
Epistaxis (%)	2 (3.3)	3 (5.0)		
Sinus orifice obstruction (%)	1 (1.7)	4 (6.7)		
Lacrimal duct injuries (%)	1 (1.7)	3 (5.0)		
Total adverse reactions (%)	5 (8.3)	14 (23.3)	5.065	0.024

## Discussion

DTRS is a medical condition characterized by nasal inflammation, presenting with primary symptoms such as nasal congestion, thick or purulent secretions, and headaches. If left untreated, DTRS can potentially impact patients' nerves and visual function, leading to adverse effects, including decreased eyesight. Additionally, it can lead to intracranial infections, manifesting as symptoms such as headaches, fever, and vomiting, thereby significantly compromising patients' overall health and disrupting their daily lives.^17^ At present, one of the primary treatment modalities for DTRS is nasal endoscopy surgery, which offers a clear view of anatomical structures and lesions, which increases the chance for improved therapeutic outcomes.^18^ However, it is essential to acknowledge that nasal endoscopy surgery may also inadvertently trigger an increase in the production of inflammatory mediators due to its invasive nature.^19^ Consequently, the supplementation of drug therapy alongside surgical intervention becomes imperative to enhance clinical efficacy. To date, limited research has explored the impact of budesonide suspension on DTRS. Therefore, in this research work, we compared the clinical efficacy of budesonide suspension in combination with surgical treatment versus surgical treatment alone.

This present study revealed no significant differences in SNOT-22 or Lund-Kennedy scores between the two groups before treatment initiation. However, following treatment, both groups exhibited a notable decrease in their respective scores, with the group receiving budesonide suspension in conjunction with PNN resection demonstrating superior efficacy compared to those receiving surgical treatment alone. Thus, the combination of budesonide suspension and surgical intervention led to a greater enhancement in patients' postoperative quality of life compared to surgical treatment alone. Additionally, the incidence of adverse reactions, including nasal adhesions, epistaxis, sinus orifice obstruction and lacrimal duct injuries, was lower in the study group compared to the control group. Furthermore, the study group displayed a significantly lower overall rate of adverse reactions. These findings underscore the enhanced efficacy of budesonide suspension when combined with surgical treatment for DTRS. Although the precise pathogenesis of DTRS remains incompletely understood, several contributing factors, including environmental, genetic, anatomical, and immune abnormalities, have been consistently identified as influencing factors. Inflammation has been confirmed as a key inducing factor in this condition.^20^ Presently, the significant role of eosinophilic infiltration in the pathogenesis of DTRS has been established.^21^ Specifically, local activation and degranulation of eosinophils lead to local epithelial damage, the production of inflammatory mediators, nasal mucosal edema, and the exacerbation of rhinosinusitis symptoms.^21^ As a chronic ailment, DTRS necessitates timely symptomatic treatment for the relief and suppression of patient symptoms. PNN resection has been substantiated as an effective treatment approach for DTRS. Thus, PNN resection can ameliorate clinical symptoms by removing a substantial portion of parasympathetic nerve fibers within the nasal cavity. This reduction in glandular secretion, inhibition of hemangiectasis, and decrease in inflammatory factor levels have been reported as key mechanisms through which PNN resection improves the condition.^6^ Notably, after PNN resection, a significant reduction in eosinophil counts within the nasal mucosa of patients with allergic rhinitis has been observed, accompanied by a marked decrease in inflammation occurrence.^22^

In addition to surgical interventions, patients with DTRS can be treated with anti-inflammatory drugs. Currently, glucocorticoids represent the primary pharmacological approach for managing DTRS. They can effectively temper the inflammatory response, reduce the release of inflammatory mediators, and facilitate the synthesis of anti-inflammatory factors through multiple mechanisms, primarily due to DTRS's increased sensitivity to glucocorticoid therapy.^23^ Additionally, glucocorticoids possess anti-biofilm properties and can facilitate the targeted delivery of medications to the afflicted mucosa within the nasal cavity or sinuses, thereby alleviating adverse reactions. An important aspect of treatment for DTRS involves the correct application of nasal glucocorticoids. It is essential to ensure that patients are well-informed and capable of properly applying these medications. Many patients struggle with the correct application of nasal glucocorticoids, leading to suboptimal drug delivery, unsatisfactory treatment outcomes, and delayed disease management.^24^ Budesonide suspension, categorized as a nebulized glucocorticoid, offers anti-inflammatory attributes and can effectively inhibit the activation of macrophages, eosinophils, and neutrophils. Notably, transnasal nebulization inhalation administration enables direct drug action on the local nasal and sinus mucosa, facilitating comprehensive drug absorption within the mucosal tissues, which amplifies the pharmacological efficacy of drugs, significantly ameliorating symptoms in individuals with sinusitis.^25^ Budesonide transnasal nebulization therapy effectively addresses the challenge of inadequate hormone delivery within the nasal cavity. Topical therapy, utilizing high-volume corticosteroid irrigation, is a viable option for DTRS patients, substantially enhancing SNOT-22 and Lund-Kennedy scores.^26^ High-volume irrigation has the advantage of precise drug distribution to the paranasal sinuses. Taken together, the methods have their own advantages and considerations. The choice between a nasal spray and nebulization often depends on the specific circumstances, including the patient's condition, the specific drug being administered, and the desired effects. Therefore, a robust patient-doctor dialogue is imperative to educate patients on the correct technique for nasal spray application. This interaction not only enhances treatment efficacy but also empowers patients to be active participants in their healthcare.

In our study, we observed that budesonide transnasal nebulization, as a form of glucocorticoid administration, offers a viable alternative, especially for patients who find it challenging to use nasal sprays correctly. The transnasal nebulization therapy provided direct and thorough drug contact with the lesions. Furthermore, nebulization facilitates aerosol penetration through the sinus orifices and sinus cavities. Due to the particulate nature of small aerosols, budesonide achieves localized deposition more readily, aided by air compression pumps, thus elevating drug concentrations and prolonging therapeutic effectiveness. Moreover, nebulization therapy garners higher patient acceptance due to its simplicity and minimal wound irritation. Thus, our combination of PNN resection with hormone transnasal nebulization significantly enhanced the SNOT-22 and Lund-Kennedy scores among DTRS patients compared to PNN resection alone, resulting in decreased adverse reactions and a more robust improvement in patients' physical and mental well-being.

However, it should also be noted that in the management of DTRS, it is important to conduct a comprehensive re-evaluation of the underlying diagnosis and to thoroughly assess any associated comorbidities before advancing to more complex treatments, including surgical interventions, especially via a multidisciplinary approach. Such a holistic approach would ensure that the treatment plan is tailored to the individual patient's needs, addressing not only the symptoms but also the root causes and related health issues, thereby contrasting with a solely surgical approach, which may not address the underlying pathology effectively. Implementing a comprehensive evaluation process can lead to more targeted and effective management of DTRS, potentially reducing the need for more invasive procedures and the risk of recurrences. In addition, it should also be noted that although combination treatment can improve clinical outcomes, this might also lead to increased risks of treatment-related complications, adverse events and financial constraints to the patients and their relatives. As a retrospective analysis, certain limitations, such as the small sample size, were inherent to this study. To further validate the findings of this investigation, future research could consider conducting large-scale, multi-center, prospective studies.

## Conclusion

In conclusion, the combination of PNN resection and hormone transnasal nebulization treatment demonstrates favorable efficacy in addressing DTRS. This therapeutic approach effectively alleviates the symptoms experienced by DTRS patients, reduces the occurrence of adverse reactions, and enhances the overall quality of life for these individuals. Hence, the adoption of PNN resection in conjunction with hormone transnasal nebulization warrants consideration and promotion within clinical practice.

## Authors’ contributions

Xin-ke Wang conceived the study. Qi-ling Zheng extracted all dada. Jia-ning Sun wrote the paper. Xin-ke Wang, Qi-ling Zheng and Jia-ning Sun undertook the statistical analyses. All authors read and approved the final manuscript.

## Funding

This work was supported by Yuyao Science and Technology Bureau Project (nº 2021YPT06).

## Availability of data and materials

The datasets used in this article are available from corresponding author on reasonable request.

## Ethics approval and consent to participate

All patients in this study signed informed consent. Besides, this study was approved by the ethics committee of Yuyao People's Hospital of Zhejiang Province (2023-03-007).

## Conflicts of interest

The authors declare no conflicts of interest.
